# Acculturation and Anger Expression Among Iranian Migrants in Germany

**DOI:** 10.3389/fpsyg.2022.715152

**Published:** 2022-03-17

**Authors:** Donya Gilan, Antonia M. Werner, Omar Hahad, Klaus Lieb, Emily Frankenberg, Stephan Bongard

**Affiliations:** ^1^Department of Psychology, Goethe-Universität Frankfurt am Main, Frankfurt am Main, Germany; ^2^Leibniz Institute for Resilience Research, Mainz, Germany; ^3^Department of Psychiatry and Psychotherapy, University Medical Center, Mainz, Germany; ^4^Department of Cardiology, Cardiology I, University Medical Center, Mainz, Germany; ^5^German Center for Cardiovascular Research (DZHK), Partner Site Rhine-Main, Mainz, Germany

**Keywords:** anger, display rules, emotion regulation, acculturation, intercultural

## Abstract

Cultural and biographical influences on the expression of emotions manifest themselves in so-called “display rules.” These rules determine the time, intensity, and situations in which an emotion is expressed. To date, only a small number of empirical studies deal with this transformation of how migrants, who are faced with a new culture, may change their emotional expression. The present, cross-sectional study focuses on changes in anger expression as part of a complex acculturation process among Iranian migrants. To this end, Iranian citizens in Iran (*n* = 61), German citizens (*n* = 61), and Iranian migrants in Germany (*n* = 60) were compared in terms of anger expression behavior and acculturation strategy (assimilation, separation, integration, marginalization) was assessed among the migrants, using the Frankfurt Acculturation Scale (FRACC). A questionnaire developed in a preliminary study was used to measure anger expression *via* subjective anger experience and anger expression within 16 hypothetical situations. Multivariate Analyses of Variance (MANOVA) revealed that Iranians and Iranian migrants reported higher anger experience ratings than Germans and directed their anger more often inward (*anger-in*). Further findings suggest that transformation processes may have affected Iranian migrants in terms of suppressed anger (*anger-in*): Iranian migrants with a higher orientation toward German culture reported lower average anger-in scores. These results suggest that there was different emotional expression among Iranian migrants, depending on their acculturation. The results provide new insight into socio-cultural and individual adjustment processes.

## Introduction

In view of increasing globalization processes and refugee movements, more and more individuals are leaving their familiar socio-cultural environment in order to establish themselves in new societies. In doing so, they go through complex acculturation processes, including psychological adaption. Specific behavior, learned within the heritage culture, is rejected (“cultural shredding”), whereas new behavior is adopted (“cultural learning”). Encounters between members of different cultures can result in “cultural conflicts,” which can be traced largely to a lack of knowledge of the values and norms for emotional expression as well as differences in motivational factors including empathy or tolerance of ambiguity of the respective groups (Berry, 2005).

Cultural and biographical influences have an impact on the expression of emotions and manifest themselves in so-called “display rules,” which determine the time, intensity, and the social situation in which an emotion is expressed (Ekman and Friesen, 1969). There is high intercultural agreement about how anger is displayed in facial expressions (Ekman, 1994). However, due to culture-specific distal and proximal factors, individuals differ in how they experience and express anger, e.g., depending on the situations which trigger the emotion (Ekman, 1972; Tavris, 1982). In previous studies, display rules for emotions were mainly examined in terms of cultural comparisons, whereby members from individualistic societies were oftentimes contrasted to those from collectivist societies. In a study by Cole et al. (2002), Euro-American children expressed anger more frequently in comparison with Nepalese children. Lewis et al. (2010) showed that Euro-American children expressed significantly more negative emotions such as anger in performance situations than did Japanese children. These remarkable findings indicate that, in collectivist cultural contexts like Nepal or Japan, the expression of self-involved negative emotions, such as anger does not seem to have an appropriate place. This may mean that children living in collectivist societies learn to suppress anger or, at least, to control it in order to uphold the harmony of the group. In the context of a more individualistic socialization, as is the case in Germany, parents convey display rules for negative emotions to children, which tends to lead to a more open form of expression. This supports the development of members of individualistic societies to view emotions as an authentic part of their personality. As a result, they are less hesitant to express negative emotions.

In terms of acculturation, the question arises as to how the processing of anger might change when individuals leave their socio-cultural environment, migrate, and adapt to a new culture. To date, few studies have dealt with the transformation of emotion expression over the course of the acculturation process. Anger was chosen as the target emotion due to its potential to create conflict. When differences in anger expression trigger conflict among members of different cultures, disputes may be related less to the cause of the anger than the manner in which it is communicated. Migrants faced with a new socio-cultural environment are frequently unable to resort to the display rules learned in their heritage culture because these can result in misunderstandings in the new cultural setting. In order to avoid such misunderstandings, they must accommodate the emotional forms of expression of the majority society. It could be argued that migrants’ preference for specific forms of emotion expression might depend on their orientation toward their heritage and receiving culture. It may also depend on individual factors and opportunities that are offered to them by their new cultural environment such as integration policy, welcome culture or attitude to diversity.

### Cultural Distance Between Iran and Germany

As highlighted above, research suggests that emotional expression differs between members from individualistic cultures and members from collectivistic cultures (Matsumoto, 1990; Matsumoto et al., 2005; Safdar et al., 2009). The investigations carried out by Hofstede (1980, 2001) and Hofstede et al. (2010) evaluating cross-cultural differences of norms and values, showed that Iran and Germany differ on various cultural dimensions, specifically in “individualism/collectivism” and in relation to “power distance,” which are of special interest for this study. Germany is classified as a relatively individualistic culture, whereas Iran is described as having a greater collectivistic orientation (House et al., 2001, 2004; Hofstede et al., 2010). In countries with an Eastern cultural background like Iran, individuals in dependent relationships stick together, i.e., have interdependencies, which is why social networks are paramount for more pronounced group cohesion. Loyalty within family contexts and other private relationships, and social harmony and group interests in general are of greater importance in Iran and other collectivistic cultures than in Germany or other individualistic countries rather associated with a more Western culture (Markus and Kitayama, 1991). The relationships of German persons are less close and there is a greater sense of self-reference: striving for individual success is viewed as a priority and conflicts are not avoided to achieve this goal. Furthermore, Germany has developed over time relatively flat hierarchies (power distance), whereas, in Iran, vertical structures are an accepted prerequisite for group functioning (Hofstede et al., 2010). Hence, there is evidence that there are large cultural differences between Iran and Germany concerning prevailing standards and values, which, among other things, is reflected in different display rules for the expression of emotions. However, changes of emotional expression like anger due to socio-cultural acculturation processes have not yet been explored in depth.

### Acculturation Processes

One of the best-known and widespread classifications of acculturation processes was created by Berry (1997, 2003) who argued that orientations (attitudes) and strategies (behaviors) result from a combination of an individual’s orientation toward the receiving culture on the one hand and the identification with the heritage culture on the other hand. The fundamental assumption is that the degree of identification with each, the heritage and the receiving culture, shapes different acculturation orientations and strategies. Migrants are faced with the question of how much they would like to maintain their original cultural identity and to which extent they would like to establish relationships with members of the receiving culture. The four possible combinations of the answers to both questions results in the following acculturation strategies: *integration*, *assimilation*, *separation*, and *marginalization*. Assimilation is defined as preferred adaptation to the receiving culture accompanied by simultaneous distancing from the culture of heritage. Separation refers to the preference of maintaining one’s the culture of heritage with little or no interest in interacting with members of the receiving culture. Integration is defined as the maintenance of the culture of heritage and establishment of contact with members of the receiving culture. Finally, marginalization identifies the loss of the culture of heritage with simultaneous isolation from the receiving culture. Modern theories specify that, within a global integration process, these four types of acculturation can differ not only between individuals but also intrapersonally, depending on the domain of life (Arends-Tóth and Van de Vijver, 2004).

Furthermore, studies indicate that transformation processes during acculturation also take place on an emotional level (Bongard at al., 2002). De Leersnyder et al. (2011) showed that migrants differ in terms of their emotional reactions depending on the extent of interaction with the majority society. The greater the interaction with the majority society, the more similar migrants’ emotional reactions were to the reactions of members of the receiving culture. Regarding the expression of emotions, this raises the question of whether an orientation toward the heritage or receiving culture or a specific acculturation strategy (Berry, 2003) has an impact on the application of new display rules. More specifically, to which extent are new display rules adopted and accepted. It is to be expected that an increasing orientation toward the receiving culture will also result in an adaptation of the receiving society’s emotional display rules.

### Objectives of the Study

The first objective of this study was to investigate within a sample of Germans, Iranians, and Iranian migrants in Germany, whether previously learned culture-specific forms of the expression of anger, which are determined by display rules (Ekman and Friesen, 1969), differ between these three groups. Following the premise of individualistic and collectivistic differences in anger expressions, it was expected that Iranians and Germans differ in their anger expression ratings. Furthermore, we expected Iranian migrants in Germany to rank in between the two national groups in terms of anger expression.

A second objective was to examine whether Iranian migrants express their anger more like Iranians or more like Germans depending on their acculturation strategy (i.e., integration, assimilation, separation, or marginalization; Berry et al., 1989). We expected Iranian migrants assigned to the assimilation acculturation strategy to differ significantly from Iranian citizens in terms of the experience and expression of anger. Conversely, it was expected that Iranian migrants assigned to the separation strategy would differ significantly from Germans in terms of both feelings and expression of anger. Accordingly, we expected to find significant differences between separated and assimilated Iranian migrants in terms of experience and expression of anger.

## Materials and Methods

### Participants and Study Procedure

Following a convenience sampling method, participants were recruited in 2012/2013 in Iran and Germany *via* various associations, educational establishments, research institutes, universities, businesses, and private households. The selection criteria were as follows: participants should be at least 18 years old and not have any mental impairment or mental disorders. The whole study was carried out with a paper-and-pencil questionnaire including measures for anger experience, anger expression, and socio-demographic information. Iranian migrants were additionally presented with questions regarding acculturation and migration history. There were two possible settings for participants who took part in this study: either the examination took place in a face-to-face setting, with participants answering the survey directly in the interviewer’s presence, or they completed the survey alone at home and sent their forms back to the study director. Participation was voluntary and anonymous, and participants did not receive monetary compensation for participating. All participants gave their informed consent regarding the study conditions at the beginning of the session. The survey was presented to Iranians in Farsi, to Germans in German. Iranian migrants could choose between Farsi and German for their questionnaire before they began to fill it out. The study was approved by the department’s ethical committee and was conducted in accordance with the Helsinki declaration on ethical principles for research involving human subjects.

### Measures

#### Feelings of Anger and Anger Expression

In a previous study, Gilan (2014) developed a questionnaire for the assessment of experienced anger and anger expression. This questionnaire contains the description of 16 hypothetical daily situations suited to induce anger in the respective protagonist. The situations represent various domains of life and include different interaction partners who are the source of possible anger.

For each situation, participants were asked to indicate how strongly they would feel anger in such a situation (*anger-reaction,* AR). *Anger-reaction* was assessed on a five-point Likert-scale (1 = “low,” 3 = “moderate,” 5 = “strong” with unlabeled intermediate points 2 and 4). In a second step, participants answered 12 items taken from the German version of the *State–Trait Anger Expression Inventory* (STAXI, Schwenkmezger et al., 1992) to report how they would manage their experienced feelings of anger in each presented situation. Four of the 12 items represented one of the three STAXI scales, respectively: *anger-in* (AI), *anger-out* (AO), and *anger-control* (AC). *Anger-in* assesses the tendency to turn anger inwards, to suppress it, i.e., to not show it. *Anger-out* measures the tendency to express anger openly, i.e., against the cause of anger. *Anger-control* reflects the tendency to avoid anger in the first place for example by calming oneself down through cognitive strategies before the feeling of anger grows. Possible responses ranged from 1 = “not at all” to 4 = “absolutely” on a four-point Likert-Scale for all three scales.

Scores were computed based on the ratings for *anger-reaction*, *anger-in*, *anger-out*, and *anger-control* in relation to each anger situation. For *anger-in*, *anger-out*, and *anger-control*, a mean score per situation, and additionally, the mean values of *anger-in*, *anger-out*, and *anger-control* over all 16 presented situations were computed. For *anger-reaction*, a mean value of the ratings based on all 16 situations was calculated since this variable was assessed by a single item.

#### Frankfurt Acculturation Scale

The *Frankfurt Acculturation Scale* (FRACC; Bongard et al., 2020) is composed of 20 statements covering the following areas: leisure activities, use of media, values, attitudes, language, social contacts, traditions, and religion. Participants rate their agreement to a statement regarding their behavior, attitude, or feelings on a seven-point Likert scale (0 = “strongly disagree” to 6 = “strongly agree”). The questionnaire contains two subscales composed of 10 items each: *orientation toward the heritage culture* (HC) and *orientation toward the receiving culture* (RC). The FRACC subscales have shown acceptable internal consistencies (HC: *α* = 0.79; RC: *α* = 0.83; Bongard et al., 2020). In the current study, the respective internal consistencies were acceptable as well, with *α* = 0.78 for HC and *α* = 0.78 for RC.

#### Socio-Demographic Questionnaire

A socio-demographic questionnaire was used to obtain personal information such as gender, age, marital status, religious affiliation, educational attainment, and occupation for the whole sample. The subsample of Iranian migrants answered additional questions regarding their migration [e.g., date of migration, reason(s) for migration, and plans of return] and acculturation (nationality of their romantic partner, extent of contact with locals, self-assessment with regard to German and/or Farsi language skills).

### Statistical Analyses

Before conducting descriptive and inferential analyses, the raw data was screened for missing data. There were only occasional missing data in the form of answers to a single questionnaire item. In these cases, the data was imputed manually with the individual’s mean value in the corresponding scale. Two cases within the migrant group were excluded as each of them did not answer one anger situation completely, leading to a migrant sample of *N* = 60 participants.

Descriptive data will be presented, including mean values and standard deviations for the total sample and each group separately regarding *anger-reaction*, *anger-in*, *anger-out*, and *anger-control*. Within the sample of Iranian migrants only, means and standard deviations for *orientation toward the heritage culture* (HC) and *orientation toward the receiving culture* (RC) will be reported for all participants in this subsample, and separately for the four acculturation orientation groups integration, assimilation, separation, and marginalization. To form these four acculturation groups, we conducted a sample-based median-split to assign the participants to the corresponding groups. Depending on the individuals’ scores on HC and RC, the sample of Iranian migrants was divided *via* the sample-driven median into subjects with “low” and “high” scores for each scale. Participants, who scored “high” on both scales were allocated to the acculturation strategy *integration*, those “high” in RC und low in HC were allocated to *assimilation*, those with “low” values in RC and “high” values in HC were allocated to *separation*, and those with “low” values on both scales were allocated to the acculturation strategy *marginalization*. At this stage, outliers were identified, and we tested, whether the data were normally distributed.

To test for significant differences between Iranians, Germans, and Iranian migrants (first study question), we compared the response variables (mean scores for overall *anger-reaction*, *anger-in*, *anger-out*, and *anger-control*) of the Iranian migrants with those of the participants of the heritage (Iranian subsample) and receiving culture groups (German subsample) by conducting a Multivariate Analysis of Variance (MANOVA).

For our second study question, we conducted a second MANOVA with the same dependent variables within the subsample of Iranian migrants (mean scores of overall anger-reaction, anger-in, anger-out, and anger-control) and the factor *acculturation orientation* (*k* = 4), testing for differences between the four acculturation groups *integration*, *assimilation*, *separation*, and *marginalization*. In both MANOVAs, required assumptions were checked prior the computation.

In order to estimate effect sizes in both of the MANOVAs, partial Eta-squared (*η*_p_^2^) will be reported and interpreted according to Cohen (1988) as follows: no effect: *η*_p_^2^ < 0.01; small effects: *η*_p_^2^ = 0.01–0.06; medium effects: *η*_p_^2^ = *0*.06–0.14; large effects: *η*_p_^2^ > 0.14. Post-hoc tests were used for significant differences between the groups in pairs with regard to the response variables.

Beyond that, we expected differences between Iranian migrants displaying a separation orientation and Germans, as well as differences between Iranian migrants with an assimilation strategy and Iranians. To test for differences, we conducted *t*-tests comparing each of these two acculturation strategy groups with both the Iranian and the German sample, in addition to the main analysis (MANOVA). We applied a Bonferroni-correction and assumed statistical significance at the *α*-level of *α* = 0.008. For these *t*-tests Cohen’s *d* is reported with *d* = 0.2 representing a small effect, *d* = 0.5 a medium effect, and *d* = 0.8 a large effect. For all analyses, IBM SPSS 24 (IBM Corp, 2016) or IBM SPSS 27 (IBM Corp, 2020) were used.

## Results

Table 1 displays the socio-demogrphic characteristics of all participants. The three sub-samples are comparable in terms of gender and age as there are no significant group differences regarding gender distribution [Kruskal–Wallis *H*(2) = 0.11, *p* = 0.95] and mean age [*F*(2, 176) = 0.04, *p* = 0.97]. Regarding marital status and education, on the descriptive level, there are some differences between the groups. The number of Iranians and Iranian migrants who were married was higher than that of German participants, yet the relative frequencies were not significantly different [Kruskal–Wallis *H*(2) = 4.38, *p* = 0.11]. In terms of highest educational level, more Iranians and Iranian migrants than Germans reported having a university degree, but overall there were no significant differences in the three groups regarding the distribution of different educational levels [Kruskal–Wallis *H*(2) = 1.88, *p* = 0.39]. Regarding reported occupational status, there were significant differences between the three groups: more Iranian people reported to be employed than Germans [Kruskal–Wallis *H*(2) = 33.59, *p* < 0.001].

**Table 1:** Socio-demographic characteristics of participants.

	Total sample	Iranians	Germans	Iranian migrants
*N*	184	61	61	62
Gender				
Female, *N* (%)	107 (58.2)	36 (59.0)	36 (59.0)	35 (56.5)
Male, *N* (%)	77 (41.8)	25 (41.0)	25 (41.0)	27 (43.5)
Age range	18–76	18–68	21–65	18–76
Mean age (SD)	36.80 (12.39)	37.08 (9.46)	36.47 (12.03)	36.85 (15.12)
Marital status				
Single, *N* (%)	43 (23.4)	14 (23.0)	14 (23.0)	15 (24.2)
In a relationship, *N* (%)	39 (21.2)	9 (14.8)	19 (31.1)	11 (17.7)
Married, *N* (%)	90 (48.9)	35 (57.4)	22 (36.1)	33 (53.2)
Separated, *N* (%)	2 (1.1)	/	2 (3.3)	/
Divorced, *N* (%)	7 (3.8)	2 (3.3)	4 (6.6)	1 (1.6)
Other, *N* (%)	2 (1.1)	1 (1.6)	/	1 (1.6)
Missing, *N* (%)	1 (0.5)	/	/	1 (1.6)
Educational background				
University degree, *N* (%)	94 (51.1)	42 (68.9)	24 (39.3)	23 (37.1)
High school graduation, *N* (%)	55 (29.9)	13 (21.3)	19 (31.1)	28 (45.2)
Mid-level high school, *N* (%)	26 (14.1)	3 (4.9)	14 (23.0)	9 (14.5)
Certificate of secondary education, *N* (%)	8 (4.3)	3 (4.9)	4 (6.6)	1 (1.6)
None, *N* (%)	1 (0.5)	/	/	1 (1.6)
Occupation				
Working, *N* (%)	107 (58.2)	50 (82.0)	41 (67.2)	16 (25.8)
In training, *N* (%)	29 (15.8)	4 (6.6)	7 (11.5)	18 (29.0)
Retired, *N* (%)	7 (3.8)	1 (1.6)	1 (1.6)	5 (8.1)
Homemaker, *N* (%)	13 (7.1)	4 (6.6)	2 (3.3)	7 (11.3)
Unemployed, *N* (%)	8 (4.3)	1 (1.6)	3 (4.9)	4 (6.5)
Other, *N* (%)	1 (0.5)	/	/	1 (1.6)
Missing, *N* (%)	19 (10.3)	1 (1.6)	7 (11.5)	11 (17.7)
Time in Germany				
Range in months				1–54
Mean time in months (SD)				18.77 (12.04)

Statistical differences between groups only for occupational status.

### Associations Between Anger-Reaction, Anger-in, Anger-out, and Anger-Control

A summary of the correlations between the anger-related variables (overall *anger-reaction*, *anger-in*, *anger-out*, and *anger-control*) is displayed in Table 2. There are small to medium associations within the anger variables. *Anger-reaction* is negatively associated with *anger-control* and positively correlated with both *anger-out* and *anger-in*. Furthermore, *anger-out* shows moderate, negative correlations with *anger-control* and a weak, positive association with *anger-in*.

**Table 2:** Intercorrelations between anger-reaction (AR), anger-control (AC), anger-in (AI), and anger-out (AO) in the total sample (*N* = 182).

	AC	AO	AI
AR	−0.25^**^	0.52^**^	0.47^**^
AC		−0.39^**^	0.09
AO			0.21^**^

** *p* < 0.001.

### Comparing Iranians, Germans, and Iranian Migrants in Germany

Table 3 presents the mean values and standard deviations of the anger variables for the three subsamples and the total sample. Values can range between 1 and 5 for *anger-reaction* and between 1 and 4 for *anger-in*, *anger-out*, and *anger-control*, in accordance with the Likert-Scales used.

**Table 3:** Means, standard deviations, and one-way analyses of variance in anger-reaction, anger-control, anger-out, and anger-in.

	Iranians (*n* = 61)	Iranian migrants (*n* = 60)	Germans (*n* = 61)	Total sample (*N* = 182)	*F*(2,179)	*p*	*η* ^2^
	*M* (SD)	*M* (SD)	*M* (SD)	*M* (SD)			
Anger reaction	3.78 (0.53)	3.68 (0.61)	3.38 (0.69)	3.61 (0.64)	7.06	<0.01	0.07
Anger-in	2.65 (0.53)	2.47 (0.49)	2.14 (0.35)	2.42 (0.51)	19.02	<0.001	0.18
Anger-out	1.90 (0.45)	1.85 (0.41)	1.87 (0.49)	1.87 (0.45)	0.18	0.83	<0.01
Anger-control	2.77 (0.53)	2.94 (0.37)	2.69 (0.36)	2.80 (0.44)	5.85	<0.01	0.06

Kolmogorov–Smirnov-tests confirmed normal distribution for all anger variables in the total sample and each subsample. Using Pillai’s trace, there was a significant effect of group on the tested dependent variables, *V* = 0.26, *F*(4, 176) = 6.69, *p* < 0.001. Even if there was no equality of the covariance matrices of the dependent variables between the groups, as shown by a significant Box test (*p* < 0.001), the group sizes were equal and therefore, Pillai’s trace can be assumed to be sufficiently robust (Field, 2013).

Several separate ANOVAs following the MANOVA revealed that the three groups differed significantly regarding *anger-reaction*, *anger-control* and *anger-in*, but not in terms of *anger-out*. While the effect can be classified as moderate regarding *anger-control* (*η*_p_^2^ = 0.06) and *anger-reaction* (*η*_p_^2^ = 0.07), group differences in *anger-in* represent a large effect (*η*_p_^2^ = 0.18).

Post-hoc test using the Games-Howell procedure showed that both Iranians and Iranian migrants reported stronger feelings of anger (*anger-reaction*) than the participating Germans. Similarly, the Iranian and Iranian migrant subsamples scored significantly higher on *anger-in* than the German subsample, in line with expectations. Furthermore, Iranian migrants scored higher on *anger-control* compared to German participants (*p* < 0.001). There were no differences regarding *anger-out* (see Table 3).

### Anger Variables Among Iranian Migrants

Before we conducted the second MANOVA, considering only the sub-sample of Iranian migrants, we assigned the participants to one of the four acculturation orientations *assimilation*, *integration*, *separation*, or *marginalization*. To this end, the median scores of the HC and RC scales (*Md_HC_* = 34, *Md_RC_* = 45) were used as cut-off scores, with participants with values of HC ≤ 34 and RC > 46 being assigned to the assimilation group, and those with values of HC > 35 and RC ≤ 45 allocated to the separation group. When HC and RC were above the median (HC > 35 and RC > 46), we assumed integration as the preferred acculturation strategy, and when both scores were below the median (HC ≤ 34 and RC ≤ 45), participants were assigned to the marginalization group. With this procedure, 38% (*n* = 23) were assigned to the separation group, 35% (*n* = 21) to the assimilation group, 17% (*n* = 10) to the marginalization group, and 10% (*n* = 6) were assigned to the integration group. Table 4 shows sample sizes, means and standard deviations of the migrant sample regarding HC and RC.

**Table 4:** Means and standard deviations of orientation toward the heritage culture (HC) and orientation toward the receiving culture (RC) of the Iranian migrant sample.

	Integrated migrants (*n* = 6)	Assimilated migrants (*n* = 21)	Separated migrants (*n* = 23)	Marginalized migrants (*n* = 10)	Total sample (*N* = 60)
Measure	*M* (SD)	*M* (SD)	*M* (SD)	*M* (SD)	*M* (SD)
HC	39.17 (3.31)	26.33 (4.65)	44.70 (5.51)	28.60 (7.52)	35.03 (10.02)
RC	48.50 (4.72)	52.95 (4.03)	38.00 (6.11)	39.60 (5.54)	44.67 (8.64)

Comparing the four groups regarding differences in *anger-reaction*, *anger-in*, *anger-out*, and *anger-control*, the multivariate analysis of variance yielded a non-significant result [Pillai’s trace, *V* = 0.27, *F*(12,165) = 1.35, *p* = 0.20]. However, the univariate ANOVAs revealed a significant group effect for *anger-in* [*F*(3, 56) = 3.27; *p* < 0.05] with a large effect size of *η*_p_^2^ = 0.15. Beyond that, the post-hoc comparison following the Games-Howell procedure revealed that the difference between *assimilation* and *marginalization* was marginally significant for *anger-in* (*p* = 0.06). Table 5 displays the means and standard deviations of all anger variables for the four acculturation groups as well as all results of the ANOVAs.

**Table 5:** Means and standard deviations of anger-reaction, anger-control, anger-out, and anger-in separately for the acculturation orientations assimilation, integration, separation, and marginalization among participating Iranian migrants (*N* = 60), Iranians, and Germans.

	Total sample (*N* = 60)	Separated migrants (*n* = 23)	Marginalized migrants (*n* = 10)	Integrated migrants (*n* = 6)	Assimilated migrants (*N* = 21)			
	*M* (SD)	*M* (SD)	*M* (SD)	*M* (SD)	*M* (SD)	*F*(3,56)	*p*	*η* ^2^
Anger reaction	3.68 (0.61)	3.71 (0.63)	3.96 (0.42)	3.52 (0.77)	3.56 (0.61)	1.11	0.353	0.06
Anger-in	2.47 (0.49)	2.64 (0.49)	2.65 (0.27)	2.26 (0.35)	2.27 (0.52)	3.27	0.028	0.15
Anger control	2.94 (0.37)	3.03 (0.40)	2.89 (0.25)	2.95 (0.45)	2.88 (0.36)	0.70	0.559	0.04
Anger-out	1.85 (0.41)	1.83 (0.34)	2.10 (0.37)	1.64 (0.53)	1.82 (0.43)	1.95	0.132	0.10

The results of the *t*-tests are summarized in Table 6. According to these tests, Iranian migrants assigned to the assimilation group and Germans did not differ significantly in terms of *anger-in* but Iranian migrants assigned to the separation group scored higher than participants of the German subsample [*t*(82) = 5.22, *p* < 0.001, *d* = 1.28]. Similarly, migrants assigned the acculturation strategy of separation did not differ from Iranians in terms of *anger-in* while assimilated migrants scored lower on *anger-in* than Iranians with a medium effect size [*t*(80) = −2.79, *p* = 0.007, *d* = 0.71]. Thus, assimilated Iranian migrants directed their anger inward to a similar extent as Germans. Similarly, Iranian migrants with a separation strategy were similar to Iranians living in their home country. In the case of *anger-control*, Iranian migrants with a separation strategy reported controlling their expression of anger more than Germans [*t*(82) = 3.78, *p* < 0.001, *d* = 0.93], while there was no significant difference between assimilated Iranian migrants and Germans.

**Table 6:** Results of several *t*-tests between separated resp. assimilated migrant samples and an Iranian sample resp. German sample.

	Separated migrants (*N* = 23–25) and Iranian (*N* = 61)				Separated migrants (*N* = 23) and German (*N* = 61)			
	*t*	df	*p*	*d*	*t*	df	*p*	*d*
Anger-reaction	−0.48	84	0.55	−0.14	2.03	84	0.05	0.48
Anger-in	−0.05	82	0.96	−0.01	5.22	82	<0.001	1.28
Anger-control	2.13	82	0.04	0.52	3.78	82	<0.001	0.93
	**Assimilated migrants (*N* = 21) and Iranian (*N* = 61)**				**Assimilated migrants (*N* = 21) and German (*N* = 61)**			
	***t***	**df**	***p***	***d***	***t***	**df**	***p***	***d***
Anger-reaction	−1.56	80	0.12	−0.40	1.08	80	0.29	0.27
Anger-in	−2.79	80	0.007	−0.71	1.11	26.62	0.28	0.34
Anger-control	0.88	80	0.38	0.22	2.13	80	0.37	0.54

## Discussion

This study examines the question of the extent to which emotionally expressive components of anger are influenced by socio-cultural changes and which role orientation toward the heritage and receiving cultures (HC, RC) plays in this process. The first objective of this study was to examine whether previously learned culturally specific forms of anger expression differ between Germans, Iranians, and Iranian migrants in Germany. It became apparent that Iranians and Germans differ from one another concerning anger expression behavior, particularly in directing the anger inwards (*anger-in*). Iranian participants reported overall higher values in *anger-in* than Germans. Additionally, Iranian participants reported slightly higher rates of anger intensity in reaction to hypothetical situations chosen to elicit anger (*anger-reaction*). There were no differences between the Iranian and the German subsamples with regard to the overt expression of feelings of anger (*anger-out*) or the control of anger (*anger-control*).

Contrary to expectations, the subsample of Iranian migrants closely resembled Iranian participants in terms of expressed anger: Iranian migrants reported a significantly higher emotional reaction to the presented anger situations and showed a stronger tendency to direct these feelings inward (anger-in) than the subsample of Germans, but they did not differ from Iranians in these measures. Regarding the question of the extent to which they controlled the experience of anger feelings and its expression (anger-control), Iranian migrants reported higher levels of anger control than both other groups. This, too, stands in contrast to the hypothesized.

The results regarding the research question support previous theories and findings according to which collectivistic cultures like Iran seek to suppress their anger and its expression and turn the negative feelings inwards. More intense experiences of anger, as were reported by the Iranian subsample and Iranian migrants, can be interpreted as potential long-term consequences of chronic anger suppression, which is discussed as a rather dysfunctional emotion regulation strategy when dealing with emotions, and may exacerbate feelings of anger in the medium- and long-term (Gross and John, 2003; John and Gross, 2004). Additionally, the influence of cultural norms on emotional communication could be mediated by the widely differing, current political and economical situations in Iran and Germany. It could be argued that people in Iran are exposed to greater stress due to Iran’s precarious economic situation (e.g., in 2019 inflation rate of 40%; The World Bank, 2020), and associated fear of unemployment and poverty, also among academics, could be reflected in a fundamentally increased willingness to express anger.

The observation that the Iranian migrant sample reported a higher tendency to control their anger compared to both the German and the Iranian sample, may be traced back to migrants’ unique status and the stressors associated with it.

The second part of this study focused on the role of acculturation orientation (i.e., integration, assimilation, separation, or marginalization; Berry et al., 1989) in anger and its expression among Iranian migrants. When comparing the four acculturation groups in terms of differences in the experience of feelings of anger and their expression, a large group effect was found for directing the anger inwards and not showing it openly. A closer look into the means and standard deviations for anger and its expression showed that Iranian migrants allocated to the assimilation group—the group that reported highest levels of adoption of German culture—were indeed similar to Germans regarding their feelings and expression of anger. Iranian migrants differed from Iranians insofar as that they showed a weaker tendency to turn their anger inward. On the other hand, Iranian migrants allocated to the separation group were similar to Iranians in Iran with regard to the extent of experiencing and expressing anger but differed from Germans in their anger expression: they showed a stronger tendency to direct their anger inward, and seek to control the experience and expression of anger more. These results suggest that migrants’ acculturation orientation is mirrored by their patterns of anger reactions and anger expression, which—in turn—may be indicative of the relinquishment and adoption of emotional display rules typical of the heritage culture and the receiving culture, respectively.

Nevertheless, these results must be interpreted with caution, as they have several methodological limitations. First, the comparatively small sample size within the Iranian migrant sample made intergroup comparisons difficult. It weakens the statistical power due to the high measurement error, which means that significant effects may be identified even with low probabilities of significant effects being present. Moreover, classifying migrants into acculturation strategy groups based on median-split is a method which has been criticized as it leads to a loss of information as well as test power (Maxwell and Delaney, 1993). We chose this method nonetheless because it was important for us to locate each participant relatively to other Iranian migrants who took part in our study. We aimed not to draw conclusions from the group assignment on the migrants’ acculturation tendency for other migrant groups in Germany. Additionally, the variance in acculturation within the subsample of Iranian migrants was very small, making the forced median- or sample-driven splitting of participants into four acculturation styles all the more artificial. However, the categories should be interpreted in relation to the specific study sample and the assignment of participants to the respective group describes their position relative to the rest of the sample but not to a norm. Therefore, the assignment quota is neither generalizable for the whole population of Iranian migrants in Germany or elsewhere nor does the size of each acculturation group reflect the “real” acculturation behavior of Iranian migrants in Germany. Iranian migrants at large can be supposed to be more strongly assimilated, compared to other migrant groups in Germany, than the median-split implies (Bongard et al., 2020). Likewise, other international studies reported that Iranian migrants seem to display a preference toward the acculturation orientation of assimilation in the Netherlands (Te Lindert et al., 2008), as well as in the United States of America (Ghaffarian, 1987). But in order to explore predictions driven from Berry’s model of acculturation in a new context, we needed the median-split to look at the four acculturation strategies. Otherwise, we would have had only the option to see the role of orientation toward the heritage culture and the receiving culture, and not their combination.

Furthermore, causal assertions about the directions of effects are limited due to the cross-sectional design, as it does not provide information regarding possible differences in emotional expression before the time of migration, which, incidentally, may have contributed to the motivation to migrate in the first place. Longitudinal studies are therefore necessary in order to test for the direction of the relationship between migration, changes in display rules, and emotional expression.

Further methodological limitations were the statistical difference between groups regarding occupational status and the method of measuring emotional expression. In our study, only hypothetical behavior was recorded by self-report, which does not have to be compatible with actual behavior, and subject to social desirability, which was not assessed. In addition, there are warranted doubts about the validity of the survey measure due to the positive correlations found between emotional regulation styles which—in theory—are mutually exclusive (i.e., anger-in and anger-out). As a result, further studies with different data sources and survey measures appear necessary to check the validity of these findings.

## Conclusion

Even when considering the limitations, the findings described in our study may stimulate further research on the effects of migration on emotional adaptation processes. Our data demonstrate cultural differences in anger experience and partly also in anger expression in terms of the direction of anger inward and the suppression or avoidance of anger as specific regulation strategies. This means that migrants, who switched from a collectivistic to an individualistic culture or, more specifically, to a culture that differs from their culture of origin in terms of display rules, are likely to encounter other styles of emotional regulation in the migration process. Accordingly, it is relevant to examine the subjective experience of migrants in further studies in order to specify how this change is perceived, judged and experienced, e.g., whether differences in display rules are consciously perceived and, if so, whether they are viewed as a stressor, a challenge or a relief.

The interplay between different display rules for emotions, i.e., due to differences in heritage and receiving culture (HC, RC), and the limited scope of action associated with the migrant status (language difficulties, uncertainty, loss of status, lack of social support, discrimination, etc.) intensify migrants’ acculturative stress and may lead to intercultural conflict within different spheres of life (work, everyday life, partnership, etc.). Therefore, knowledge of the emotional processing mechanisms of migrants with different cultural backgrounds could improve intercultural communication and cohabitation within a pluralistic society.

## Data Availability Statement

The raw data supporting the conclusions of this article will be made available by the authors, without undue reservation.

## Ethics Statement

The studies involving human participants were reviewed and approved by the Ethics Committee of Department of Psychology and Sports Science from the Johann Wolfgang Goethe-Universität Frankfurt am Main. The patients/participants provided their written informed consent to participate in this study.

## Author Contributions

DG and SB: study concept and design. DG, AMW, OH, KL, EF, and SB: acquisition, analysis or interpretation of data, and revision for important intellectual content. DG, AMW, and EF: drafting of the manuscript. DG and AMW: statistical analysis. All authors contributed to the article and approved the submitted version.

## Conflict of Interest

The authors declare that the research was conducted in the absence of any commercial or financial relationships that could be construed as a potential conflict of interest.

## Publisher’s Note

All claims expressed in this article are solely those of the authors and do not necessarily represent those of their affiliated organizations, or those of the publisher, the editors and the reviewers. Any product that may be evaluated in this article, or claim that may be made by its manufacturer, is not guaranteed or endorsed by the publisher.
